# Perceived Versus Actual Time of Prehospital Intubation by Paramedics

**DOI:** 10.5811/westjem.18400

**Published:** 2024-06-20

**Authors:** Daniel Shou, Matthew Levy, Ruben Troncoso, Becca Scharf, Asa Margolis, Eric Garfinkel

**Affiliations:** * Johns Hopkins University School of Medicine, Department of Emergency Medicine, Baltimore, Maryland; † Howard County Department of Fire and Rescue Services, Office of the Chief Medical Officer, Marriottsville, Maryland

## Abstract

**Introduction:**

Situational awareness is essential during emergent procedures such as endotracheal intubation. Previous studies suggest that time distortion can occur during intubation. However, only in-hospital intubations performed by physicians have been studied. We aimed to determine whether time distortion affected paramedics performing intubation by examining the perceived vs actual total laryngoscopy time, defined as time elapsed from the laryngoscope blade entering the mouth until the endotracheal tube balloon passes the vocal cords.

**Methods:**

For this retrospective study we collected prehospital intubation data from a suburban, fire department-based emergency medical services (EMS) system from January 5, 2021–May 21, 2022. The perceived total laryngoscopy time was queried as a part of the electronic health record. Video laryngoscopy recordings were reviewed by a panel of experts to determine the actual time. Patients >18 years old who underwent intubation by paramedics with video laryngoscopy were included for analysis. The primary outcome was the difference between actual and perceived total laryngoscopy time. Secondary analysis examined the relationship between high time distortion, defined as the highest quartile of the primary outcome, and patient age, paramedic years of experience, perceived presence of difficult anatomy, excess secretions, use of rapid sequence intubation, and multiple intubation attempts. We conducted descriptive analysis followed by logistic regression analysis, chi-square tests, and Fisher exact tests when appropriate.

**Results:**

A total of 122 intubations were collected for analysis, and 10 were excluded due to lack of video recording. Final analysis included 112 intubations. Mean actual laryngoscopy time was 50.0 seconds (s) (95% confidence interval [CI] 43.7–56.3). Mean perceived laryngoscopy time was 27.8 s (95% CI 24.7–31.0). The median difference between actual and perceived time was 18 s (interquartile range 6–30). We calculated high time distortion as having a difference greater than 30 s between actual and perceived laryngoscopy time. None of the secondary variables had statistically significant associations with high time distortion. Overall, we show that the paramedic’s perception of total laryngoscopy time is significantly underestimated even when accounting for paramedic experience and perceived airway difficulty.

**Conclusion:**

This study suggests that time distortion may lead to an unrecognized prolonged procedure time. Limitations include use of a convenience sample, small sample size, and potential uncollected confounding variables.

Population Health Research Capsule What do we already know about this issue?

*Elevated stress and an increased cognitive load during intubations can reduce the ability of clinicians to accurately determine the passage of time.*

What was the research question?

*Among paramedics, is there a difference between perceived vs actual total laryngoscopy time (TLT)?*

What was the major finding of the study?

*Perceived TLT (26.8 seconds, 95% CI 23.7–29.8) was significantly lower than actual TLT (44.6 seconds, 95% CI 41.2–48.1).*

How does this improve population health?

*The identified time differences offer guidance for educational and procedural interventions with the goal of improving clinical outcomes for patients.*

## INTRODUCTION

Effective airway management is a critical prehospital intervention, and endotracheal intubation (ETI) has long been considered an essential paramedic skill to manage a patient with an unstable airway or ineffective breathing. While ETI is potentially lifesaving, the procedure can quickly become harmful if hypoxia develops during laryngoscopy.^1^ Although preoxygenated healthy patients may have a safe apnea time up to 10 minutes, the most likely patient to require prehospital ETI is a patient in extremis or cardiac arrest for which there is minimal literature regarding safe procedure time.^2^ The moribund patient likely already has abnormal oxygenation and ventilation, which further complicates intubation, as any amount of apnea time could worsen the patient’s condition. First-pass success and a short procedure time is the key to minimizing adverse events during intubation; however, the ability of paramedics to maintain awareness of the elapsed time during intubation is unknown.^3^^,^^4^

Anesthesia literature and high-profile events demonstrate that even expert clinicians can suffer from cognitive errors during intubation that cause an unrecognized prolonged procedure time and a poor patient outcome from hypoxia.^5^^,^^6^ Studies of healthcare workers suggest that stressful situations, such as those during an intubation or resuscitation, diminish the ability to accurately identify the passage of time.^7^^,^^8^ A study of emergency physicians found that the estimated time to intubation was significantly less than the actual procedure time.^9^ However, the generalizability of these studies to clinicians operating outside the hospital environment is unknown. To date, there has been a paucity of studies examining the ability of emergency medical services (EMS) professionals to estimate time during stressful situations in the prehospital environment.

We sought to examine the perceived vs actual total laryngoscopy time (TLT) during prehospital intubation performed by paramedics in a countywide EMS system. This information can be used to inform future best practices for prehospital intubation.

## METHODS

We performed a retrospective review comparing actual TLT vs perceived TLT among a convenience sample of all patients intubated with video laryngoscopy in the prehospital setting from January 5, 2021–May 21, 2022 at a single, combined fire and EMS department. Total laryngoscopy time was defined as time elapsed from the laryngoscope blade entering the mouth until the endotracheal tube balloon passed the vocal cords.

Patients were intubated by firefighter paramedics from Howard County Department of Fire & Rescue Services (HCDFRS). All firefighter paramedics are licensed in the state of Maryland and maintain active certification with the National Registry of Emergency Medical Technicians. The HCDFRS is a combined career-volunteer department with over 900 career and volunteer personnel. The department receives over 30,000 EMS calls per year and serves about 325,000 residents in Howard County, MD. The department is comprised of 14 stations, all of which are staffed with career personnel and six are supplemented by volunteer crews. The department operates both Basic Life Support and Advanced Life Support (ALS) transport units staffed with a minimum of two EMT-B personnel and at least one paramedic, respectively. Three paramedic duty officers (MDO) operating in fly cars provide daily operational supervision of EMS operations, incident command, and additional ALS support to crews dispatched on high-complexity calls.

Each ALS unit carries medical equipment that is standardized across the department. For intubations, the department provides video laryngoscopes (UE Scope 2, UE Medical Devices, Inc, Newton, MA) in addition to a standard complement of conventional, non-video laryngoscopes and rescue airway devices such as a bougie and supraglottic airway device (i-gel, Intersurgical, Ltd, Rugby, United Kingdom). Airway management procedures and protocols are outlined in a departmental general order. The general order recommends the use of video laryngoscopy as the preferred method of laryngoscopy. If video laryngoscope equipment is not available or if the clinical circumstances dictate, the intubation may be performed with direct laryngoscopy. Immediately following a call during which video laryngoscopy was performed, the MDO will immediately conduct a debriefing of the procedure with the responding crew and download the video for internal departmental quality assurance. In situations where oxygenation and ventilation cannot be adequately performed, an emergency needle cricothyroidotomy may be performed.

Paramedic intubation competency is assessed during yearly continuing education, which includes a one-hour airway lecture led by an EMS physician followed by a skills assessment. Paramedics who are internally credentialed to perform rapid sequence intubation (RSI) are required to attend at least one cadaver lab every year, as well as monthly RSI debriefs led by an EMS physician. The cadaver lab and debriefs are optional for non-RSI credentialed paramedics.

We conducted our retrospective chart review, data collection, and analysis following best practice methodologic standards for health record review.^10^ Following each intubation, the intubator must complete an “airway form,” which includes a question asking the paramedic to estimate the intubation procedure time. If the paramedic conducted multiple intubation attempts to successfully intubate the patient, the paramedic was asked to estimate the procedure time for each intubation attempt. The perceived TLT was then retrieved from the electronic health record. Actual TLT was determined by consensus from a trained panel of experts blinded to the study objectives who reviewed prehospital video laryngoscopy recordings obtained from the video laryngoscope. The expert panel consisted of two board-certified emergency physicians with subspecialty certification in EMS, an EMS fellow, two paramedic fire officers, one paramedic field supervisor, a quality improvement officer, and two field paramedics. If the patient was successfully intubated multiple times by the paramedic, only the first intubation was included in the dataset. The panel also collected information and came to a consensus on several variables that may have affected the airway procedure time. We then compiled all data into a dataset. Patients in which data was incomplete were excluded from the dataset prior to analysis.

Inclusion criteria consisted of all patients ≥18 years who were intubated by a paramedic with video laryngoscopy. The primary outcome was the difference between actual TLT and perceived TLT. Secondary analysis examined the relationship between high time distortion and secondary variables including patient age, paramedic years of experience, perceived presence of difficult anatomy, excess secretions, and the use of RSI. We used the highest quartile of the primary outcome as the cut-off point to define cases with high time distortion. We calculated the mean, median, and interquartile range (IQR) to provide initial descriptive analysis of the data. Outliers were defined as values that were greater than 150% of the IQR. We used the paired *t*-test to compare the difference between actual and perceived TLT, excluding any identified outliers. Logistic regression analysis, chi-square tests, and Fisher exact tests were used when appropriate to examine the relationship between high time distortion and secondary variables. We conducted all data analysis using STATA version 17, (StataCorp LLC, College Station, TX).

This study was approved by the Johns Hopkins Medicine Institutional Review Board (reference number IRB00319716).

## RESULTS

During the defined study period, a total of 122 intubations were conducted by the department, and all attempts involved the use of video laryngoscopy. No attempts used backup airway methods. Among these attempts, 112 met inclusion criteria. Ten intubations were excluded due to lack of available video recording. Table 1 demonstrates call location and ultimate disposition of the patient at the end of the call.

**Table 1.** Demographics and characteristics of patients intubated.

Characteristics	Overall (n = 112)
Age, mean (years)	61.9
Male gender (%)	62 (55%)
Call location	
Home/other residence (%)	77 (69%)
Public (%)	21 (19%)
Healthcare facility (%)	11 (10%)
Other (%)	3 (2%)
Patient disposition	
Transferred to hospital care (%)	95 (85%)
Pronounced deceased on scene (%)	17 (15%)

Patients were intubated due to cardiac arrest in 84% (94/112) of cases. Rapid sequence intubation occurred in 15% (17/112) of patients. Time range of attempts was 19–300 seconds (s), with 83% (93/112) taking 60 s or less. First-pass success was 83% (93/112) with an average time of 47.5 s (19–300). Of the attempts that took longer than a minute, the average TLT was 100.4 s. Unsuccessful attempts took an average of 62.5 s (24–120) (Table 2). Paramedics intubating had an average of 10.7 years of experience in the department and average 2–3 intubations per year.

**Table 2.** Actual intubation average time and range broken down by different groups.

Intubation sample	Average (seconds)	Range (seconds)
Overall intubation time (n = 112)	50.0	19–300
First pass – successful intubation time (n = 93)	47.5	19–300
First pass – unsuccessful intubation time (n = 19)	62.5	24–120
Total laryngoscopy time for attempts ≤60 seconds (n = 93)	39.7	19–60
Total laryngoscopy time for attempts >60 seconds (n = 19)	100.4	61–300
Rapid sequence intubation performed (n = 17)	56.2	20–120

The mean actual TLT was 50.0 s (95% confidence interval [CI] 43.7–56.3),and the mean perceived TLT was 27.8 s (95% CI 24.6–31.0). After excluding nine identified outliers the mean actual TLT was 44.6 s (95% CI 41.2–48.1), and the mean perceived TLT was 26.8 s (95% CI 23.7–29.8). The differences in means and medians between actual and perceived TLT were 17.9s (95% CI 14.5–21.2) and 18.0 s (IQR 6–29), respectively (Table 3).

**Table 3.** Comparison of mean and median actual vs perceived total laryngoscopy time among all included intubations (n = 103), excluding 9 outliers.

Sample	Actual TLT	Perceived TLT	Difference	*P*-value
All intubations				
Mean (95% CI)	50.0 s (43.7–56.3)	27.8 s (24.6–31.0s)	22.2 s (15.5–28.9)	<0.001
Median [IQR]	43.0 s [31.0–57.5]	20.0 s [15.0–30.0]	18.5 s [6–30]	<0.001
				
Excluding outliers				
Mean (95% CI)	44.6 s (41.2–48.1)	26.8 s (23.7–29.8)	17.9 s (14.5–21.2)	<0.001
Median [IQR]	43.0 s [31.0–56.0]	20.0 s [15.0–30.0]	18.0 s [6–29]	<0.001

*TLT*, total laryngoscopy time; *s*, seconds; *IQR*, interquartile range; *CI*, confidence interval.

We calculated high time distortion as having a difference greater than 29 s between actual TLT and perceived TLT. Patient age, paramedic years of experience, the use of RSI, presence of excess secretions or difficult airway anatomy, and multiple intubation attempts showed no statistically significant association with high time distortion (Table 4).

**Table 4.** Univariate logistic regression of patient and paramedic variables associated with having high time distortion. (excluding 9 outliers).

Intubation variable	OR (95% CI)	*P*-value
Patient age	0.98 (0.96–1.01)	0.15
Paramedic years of experience	0.94 (0.88–1.00)	0.06
Difficult anatomy	0.39 (0.04–3.25)	0.38
Excess secretions	0.38 (0.12–1.22)	0.11
RSI	1.24 (0.35–4.31)	0.74
Repeat attempts	2.05 (0.61–6.83)	0.24

*CI*, confidence interval; *RSI*, rapid sequence intubation.

## DISCUSSION

To our knowledge, this is the first study to investigate situational awareness of paramedics during prehospital intubations. Overall, our data shows that our paramedics had a first-pass success rate of 83% when using video laryngoscopes compared to a historic first-pass rate of 51% and overall intubation success rate of 61% in 2009, prior to the introduction of video laryngoscopes in the department.^11^ Our reported first-pass success rate is higher compared to that of other systems, such as the Seattle Fire Department (63%),^12^ and from large, multicenter studies such as the Pragmatic Airway Resuscitation Trial (51%)^13^; however, accurate comparison of first-pass rates between our cohort and that of other departments may be complicated by our relatively smaller sample size and differences in departmental protocol and training.

Our data shows an average paramedic-perceived TLT that was significantly lower than the measured TLT. This result is similar to that of previous studies of stressful situations conducted in a hospital setting. One study, following intern physicians, resident physicians, and nurses during in-hospital cardiopulmonary resuscitation simulations, found that clinicians underestimated cardiac arrest duration by 22.5 s when asked during the simulation.^14^ A similar underestimation was found in physicians and nurses during neonatal resuscitation simulations when asked to estimate time from birth to several checkpoint interventions.^8^ Finally, a study of emergency physicians examining time perception in actual emergency department RSIs found a significant underestimation of procedure time, 23 vs 45.5 s. This study also found that accuracy in determining the time elapsed worsened as more time passed during intubation.^9^

The clinical implication of underestimating elapsed time during intubation is potentially lethal. Even with preoxygenation, it takes just 10 minutes for the pulse oximetry to drop below 80% in a healthy, non-obese adult. However, a critically ill patient is more likely to have the presence of shunting, increased metabolic demand, anemia, volume depletion, and decreased cardiac output, all of which have been shown to reduce both oxygen storage in the lungs and safe apnea time.^15^ This effect would be reasonably amplified even further in cardiac arrest patients with a prolonged down time. Our results showed a median time underestimation of 18 s compared to the actual TLT. While it is not clear whether an overall difference of 18 s is significant, any time dilation could result in a longer than expected apnea time and a poor clinical outcome.

We sought to explore variables that could affect perception of intubation time such as presence of a difficult airway, RSI, past paramedic experience, and repeated intubation attempts. However, these variables were not associated with a statistically significant high time difference. Future research is required to determine which factors, if any, influence accuracy of time perception.

Current education and the body of literature emphasize first-pass success as the benchmark of a successful intubation. Multiple studies have shown an association with an increased risk of adverse events with each successive intubation attempt. However, overemphasis on first-pass success may lead to a fixation on avoiding a second intubation attempt at the cost of a prolonged procedure time and hypoxia. Monitoring for hypoxia during intubation of a critically ill patient poses a significant challenge as pulse oximetry is unlikely to be reliable secondary to poor perfusion. To address this, we propose modifying the current paradigm of a successful intubation from one that emphasizes first-pass success to one that emphasizes overall time awareness with a low threshold for recognizing and aborting a prolonged intubation attempt. Currently, the goal maximum time for a prehospital intubation is not established. In our dataset, 83% of intubation attempts were performed within one minute and attempts that lasted longer than a minute required another 40 s of procedure time, on average. The HCDFRS has implemented a maximum of 60 s for an intubation attempt given these results and the simplicity of remembering one minute per attempt. However, we acknowledge that more research is required to identify a safe maximum prehospital intubation time and that the time threshold may vary with different clinical presentations.

We propose introducing and emphasizing time awareness in prehospital intubation protocols to avoid task fixation. This modification was included in the American Society of Anesthesiologists Difficult Airway Management Guideline in 2022^5^ and has been incorporated into the department’s most recent airway management protocol. Interventions could include those already used in aviation to reduce task fixation, such as the use of checklists, closed-loop communication, and optimized data presentations.^16^^,^^17^ Additional interventions can focus on paramedic education. Initial certifying classes and continuing medical education can emphasize time awareness during intubations through focused didactics and frequent, high-fidelity simulations that emphasize the use of an airway algorithm and promote a low threshold for aborting an intubation attempt and moving to a backup airway method. Future research is required to assess the efficacy of these interventions in the prehospital environment and establish the ideal maximum procedural time for ETI.

## LIMITATIONS

Our study has significant limitations. First, we used a convenience sample of available prehospital intubations at a single site in which video laryngoscopy data was readily available. As such, our sample size is small, and we did not conduct a formal sample size calculation for this study. The ability of our conclusions to be generalized to other systems is limited and will warrant an additional, more robust study with more comprehensive sampling. Second, the data is reliant on the paramedic documenting their perceived intubation time during completion of the prehospital care report (PCR). While the department emphasizes completing the PCR upon completion of the patient transport, the paramedic has up to 24 hours to complete their it, which may affect the paramedic’s ability to accurate remember the perceived procedure time.

Additionally, this study evaluated only video laryngoscopy. Due to difficulties inherent with retrospective chart review, it was not possible to evaluate pulse oximetry or clinical status at time of intubation attempt; thus, it is not known whether there was an actual difference in rates of hypoxia with longer intubation times, although previous literature would support this assumption. This will warrant additional studies incorporating clinical data for patients who are intubated. Finally, not all possible secondary variables that may affect perception of intubation time were captured or analyzed by this study. Other variables that possibly warrant additional investigation include patient gender, indication for intubation, and estimated patient weight, among others.

## CONCLUSION

In this single-site study, the total time for video laryngoscopy intubation was significantly longer than perceived by the intubating paramedic. Emphasis should be placed on limiting the intubation time to avoid potentially catastrophic desaturation events.
